# Single-cell RNA sequencing reveals *in vivo* osteoimmunology interactions between the immune and skeletal systems

**DOI:** 10.3389/fendo.2023.1107511

**Published:** 2023-03-27

**Authors:** Shengran Wang, Jonathan Greenbaum, Chuan Qiu, Yun Gong, Zun Wang, Xu Lin, Yong Liu, Pei He, Xianghe Meng, Qiang Zhang, Hui Shen, Krishna Chandra Vemulapalli, Fernando L. Sanchez, Martin R. Schiller, Hongmei Xiao, Hongwen Deng

**Affiliations:** ^1^ Tulane Center of Biomedical Informatics and Genomics, Deming Department of Medicine, Tulane University School of Medicine, Tulane University, New Orleans, LA, United States; ^2^ Xiangya School of Nursing, Central South University, Changsha, China; ^3^ Department of Endocrinology and Metabolism, The Third Affiliated Hospital of Southern Medical University, Guangzhou, China; ^4^ Center for System Biology, Data Sciences and Reproductive Health, School of Basic Medical Science, Central South University, Changsha, China; ^5^ Center for Genetic Epidemiology and Genomics, School of Public Health, Medical College of Soochow University, Suzhou, China; ^6^ College of Public Health, Zhengzhou University, High-Tech Development Zone of States, Zhengzhou, China; ^7^ Department of Orthopaedic Surgery, Tulane University School of Medicine, Tulane University, New Orleans, LA, United States; ^8^ Nevada Institute of Personalized Medicine, University of Nevada Las Vegas, Las Vegas, NV, United States; ^9^ Institute of Reproductive and Stem Cell Engineering, School of Basic Medical Science, Central South University, Changsha, China; ^10^ Center of Reproductive Health, School of Basic Medical Science, Central South University, Changsha, China

**Keywords:** single-cell RNA sequencing, ligand-receptor, osteoimmunology, cell-specific network, LASSO

## Abstract

**Background:**

While osteoimmunology interactions between the immune and skeletal systems are known to play an important role in osteoblast development, differentiation and bone metabolism related disease like osteoporosis, such interactions in either bone microenvironment or peripheral circulation *in vivo* at the single-cell resolution have not yet been characterized.

**Methods:**

We explored the osteoimmunology communications between immune cells and osteoblastic lineage cells (OBCs) by performing CellphoneDB and CellChat analyses with single-cell RNA sequencing (scRNA-seq) data from human femoral head. We also explored the osteoimmunology effects of immune cells in peripheral circulation on skeletal phenotypes. We used a scRNA-seq dataset of peripheral blood monocytes (PBMs) to perform deconvolution analysis. Then weighted gene co-expression network analysis (WGCNA) was used to identify monocyte subtype-specific subnetworks. We next used cell-specific network (CSN) and the least absolute shrinkage and selection operator (LASSO) to analyze the correlation of a gene subnetwork identified by WGCNA with bone mineral density (BMD).

**Results:**

We constructed immune cell and OBC communication networks and further identified L-R genes, such as JAG1 and NOTCH1/2, with ossification related functions. We also found a Mono4 related subnetwork that may relate to BMD variation in both older males and postmenopausal female subjects.

**Conclusions:**

This is the first study to identify numerous ligand-receptor pairs that likely mediate signals between immune cells and osteoblastic lineage cells. This establishes a foundation to reveal advanced and in-depth osteoimmunology interactions to better understand the relationship between local bone microenvironment and immune cells in peripheral blood and the impact on bone phenotypes.

## Introduction

The immune and skeletal systems are closely linked through various physiological and pathological conditions (1, 2). Osteoimmunology interactions are involved in both bone microenvironment and peripheral circulation (1, 2). For example, activated monocytes stimulating the expression of Runx2 was critical in differentiation bone marrow-derived mesenchymal stem cells (BMSCs) into the osteoblast lineage for bone fracture repair (3, 4). The absence of neutrophils induces IL-17-driven inflammatory bone loss damaging to bone tissue (5). In addition, postmenopausal osteoporosis has been described as an inflammatory disease, as immune cells were identified as key players in the onset of osteoporosis (6, 7). The current research used single-cell RNA sequencing (scRNA-seq) data from human femoral head to explore the *in vivo* osteoimmunology communications of osteoblastic lineage cells (OBCs) with bone microenvironment immune cells. We also used scRNA-seq data and bulk transcriptomes data from peripheral blood monocytes of both male and female human subjects to reveal the osteoimmunology effect of circulating immune cells on bone health.

Previous researches have explored the heterogeneity among OBCs and microenvironment immune cells (8, 9), including our previous study which provided the first unbiased examination of the cellular landscape of freshly collected bone samples from human femoral head *via* scRNA-seq (10, 11). Although the cellular interaction between OBC and the immune system has been looked at extensively in mice, the related studies are much more uncommon in humans. CellphoneDB is a widely used cell communication software based on a public repository of ligands-receptors with complex information (12). CellChat is a classical software with pathway and multiple analysis tools through social network, pattern recognition and manifold learning (13). So systemic analysis of cell interactions can be explored by using these two complementary approaches.

In adult peripheral skeleton, peripheral blood monocytes are the sole source of osteoclast precursors which are involved in the bone resorption (by osteoclasts)-formation (by osteoblast) homeostasis (14–17). This homeostasis state is crucial for keeping the normal bone mineral density (BMD) level and is related to metabolic bone diseases like osteoporosis (18). Investigating the correlations of gene interactions with BMD may contribute to a better understanding of the osteoimmunology process of monocyte. So, we applied multiple approaches such as cell-specific network (CSN) (19) and Least absolute shrinkage and selection operator (LASSO) (20) analysis to assess gene interactions in expression profiles of circulating monocyte from male and female samples in the current study. CSN analysis allows construction of separate gene regulatory networks for individual RNA-seq samples, thereby enabling identification of multiple genes and their expression correlations based on different sample status such as age and BMD level (19). LASSO is a machine learning method that performs both variable selection and regularization to enhance the interpretability and accuracy of the prediction model (20). LASSO has been used to select prognosis/clinical character-associated genes and avoid overfitting in expression profiles of different diseases (21–23).

Here, we constructed OBC and bone microenvironment immune cell communication networks, and further identified L-R genes with ossification related functions. For circulating immune cells, CSN and LASSO analysis revealed a monocyte subtype (Mono4) related subnetwork may be associated with BMD levels in older males and postmenopausal females respectively. Our findings provide a resource of immune-skeletal system interactions, which may contribute to understanding characteristic gene’s osteoimmunology functions in skeletal physiological and pathological processes.

## Method

### Study population and bone scRNA-seq data sources

We visualized all the datasets with the sample origin, number of cells, and sequencing methodology in **Table 1**. As described in our recent study (10), the study subject was a 31-year-old male patient who was diagnosed with osteoarthritis and osteopenia. After cell digestion, a part of the cell mixture were collected as “microenvironment cells (11)” (microenvironment dataset), and the rest of the cells were used for osteoblast (OB) sorting (10) (OB sorting dataset). Detailed information on study population and bone scRNA-seq data sources were shown in **Table 1**.

**Table 1:** Detailed information on sample origin, number of cells, and sequencing methodology of each dataset.

Dataset	Sample origin	Number of cells/samples	Sequencing methodology
**Microenvironment dataset**	Femur head-derived bone tissue	8952 cells	scRNA-seq
**OB sorting dataset**	Femur head-derived bone tissue	8728 cells	scRNA-seq
**CCA integration dataset**	Femur head-derived bone tissue	17680 cells	scRNA-seq
**Blood monocytes dataset**	Peripheral blood monocytes	339 cells	scRNA-seq
**Male monocytes dataset**	Peripheral blood monocytes	944 samples	RNA-seq
**Female osteoporosis dataset**	Peripheral blood monocytes	80 samples	mRNA transcriptome array

### Study population and peripheral blood monocytes (PBMs)-related datasets

The scRNA-seq data of 339 blood monocytes were obtained from GEO database with GEO Series GSE94820 (24). In this dataset, peripheral blood mononuclear cells were isolated by FACS to exclude cells expressing markers of B, T, and NK cells and sampled LIN–CD14^lo/++^ cells for monocytes. Four monocyte subtypes were defined in the clustering analysis through Seurat R package (24).

PBMs for the RNA-seq analysis were isolated from 944 male subjects (ethnicity: Caucasian (572), African-American (371) and Hispanic (1); age: 20-64) after whole-body BMD (WB-BMD) measurements. Detailed characteristics of subjects are shown in **Table S1**. These subjects were recruited from the Louisiana Osteoporosis Study (LOS) (25–28). The Institutional Review Boards of Tulane University approved the study. Written informed consent was obtained from all participants before inclusion in the study. The WB-BMD (g/cm^2^) of each subject was measured using a Hologic dual energy x-ray absorptiometer (DXA) scanner (Hologic Corp., Waltham, MA). The machine was calibrated daily. PBMs were isolated from whole blood using a Monocyte Isolation Kit II (Miltenyi Biotec Gmbh, Bergisch Glagbach, Germany). Next, total RNA from monocytes was extracted using the AllPrep RNA Universal Kit (Qiagen, USA) following the manufacturer’s protocol and kept at -80°C until further use. After quality control, mRNA sequencing (RNA-seq) libraries were prepared following the Illumina’s TruSeq-stranded-total-RNA-sample preparation protocol. RNA libraries were sequenced on the Illumina’s NovaSeq 6000 sequencing system and generated paired-end reads. The FPKMs were calculated by StringTie (29) to evaluate the expression levels of genes in each sample. MuSiC R package (30) was used to perform deconvolution analysis based on monocyte scRNA-seq data through the package reference manual (https://github.com/xuranw/MuSiC).

The female osteoporosis mRNA transcriptome array data were obtained from 20 postmenopausal and 20 premenopausal subjects with low BMDs, and 20 postmenopausal and 20 premenopausal subjects with normal BMDs (GEO Series GSE56815) (14, 31). These data were derived from circulating monocytes isolated with a Monocyte-Negative Isolation Kit (Miltenyi Biotec) and were tested on the Affymetrix Human Genome U133A Array platform. The expression matrix has been normalized by using the RMA (robust multiarray average) method through the Bioconductor’s Oligo package.

### Integration of OBCs and bone microenvironment cells

We integrated the microenvironment dataset and OB sorting dataset by CCA using Seurat R package (32). The CCA method identified shared correlation structures across different datasets by finding linear combinations of the features with large correlation to overcome batch effect. After CCA integration, uniform manifold approximation and projection (UMAP) were used for dimension reduction. Cell clustering results were visualized in a two-dimensional panel by using DimPlot function in Seurat R package.

### Single-cell trajectory construction

We reconstructed the single-cell developmental trajectories in pseudo-time order by using Monocle 2 R package (v2.14.0) to discover developmental transitions of OBC. We used “reduceDimension” function for the dimension reduction and “orderCells” function for the cell ordering. Next, “DDRTree” and “UMAP” were applied to reduce dimension and these results were used for cell trajectory visualization.

### Gene enrichment analysis

To identify the significantly enriched pathways of cell trajectory related genes or significant L-R gene pairs, we used clusterProfiler R package to perform Gene Ontology (GO) enrichment analysis. Only terms showing adjusted p-values less than 0.05 (adjusted for multiple testing by using the Benjamini-Hochberg (BH) method) were considered as significantly enriched.

Metascape were applied for pathway enrichment analysis in KEGG and Wiki database. Metascape utilizes the well-adopted hypergeometric test and BH p-value correction algorithm to identify significantly enriched terms (adjusted p< 0.05). Next, pairwise similarities between any two terms were computed based on a Kappa-test score and a 0.3 similarity threshold was applied to get separate term clusters. Metascape chose the most significant (lowest p-value) term within each cluster to represent the cluster. Term networks were created by the representing terms as nodes and the Kappa similarities above 0.3 between pairs of nodes as edges in the Cytoscape software (v.3.9.1).

### Analysis of L-R communication

Cell communication analysis in the current research was based on complementary tolls of CellPhoneDB (12) and CellChat (13). Input for the ligand receptor analysis (both CellPhoneDB and CellChat) are cell-gene expression and cell type information matrix. These two approaches were different in data sources and analysis methods. Compared with CellChat, CellPhoneDB included more information about protein complex (**Table S2**). L-R repository used in CellPhoneDB was also larger than CellChat. On the other hand, CellChat provides unique pathway-based L-R sources. It also has multiple calculation methods regarding cofactors, mediators and pattern-based analysis tools for discovery of novel functional intercellular communications. Compared with CellChat, CellPhoneDB included more information about protein complex with a larger L-R repository (**Table S2**), while CellChat provides a unique source regarding pathway, cofactors, mediators and pattern-based analysis tools for discovery of novel functional intercellular communications.

First, we used CellPhoneDB (12) to systematically analyze the cell communication network between OBCs and bone microenvironment cells. CellPhoneDB is a public repository of ligands-receptors that considers interacting partners as binary interactions and calculates the average log gene expression level and communication significance of each known L-R pair. To perform statistical inference of L-R specificity, a null distribution for each L-R pair mean was generated by random permutation (1,000 times by default) first. The p-value for the likelihood of cell-type specificity of a given L-R pair was calculated by the proportion of the means which are ‘as or more extreme’ than the actual mean. Predicted interaction L-R pairs with p-values< 0.05 were considered as significantly differentially expressed in one cell pair compared with other cell pairs as defined in the original analyses (12).

Next, the intercellular communication pathways were analyzed by CellChat (13), a public knowledge base of ligands, receptors, cofactors, and their interactions with pathway annotation. CellChat identified the differentially expressed ligands and receptors in each cell type and clustered multiple communication patterns of different cell populations and pathways through social network analysis tool, pattern recognition methods and manifold learning approaches (13). Such analyses enable identification of the specific signaling roles played by each cell population, as well as the discovery of novel functional intercellular communications in certain cell types.

### Construction of co-expression modules and subnetwork identification

The weighted gene co-expression network analysis (WGCNA) (33) was used to identify functional gene modules. The soft thresholding power β = 14 was selected to amplify the expression differences and get a scale-free topology in the co-expression network. We set the minimum module size to be 30 genes. Each module was represented by its eigengene which was defined as the first principal component of a given module. The average gene significance (GS) was defined as the correlation between module eigengenes and phenotypes. There were five phenotypes involved in the current analysis including WBTOT_BMD and cell type proportions of four monocyte subtypes (Mono1, Mono2, Mono3, and Mono4). Module membership (MM) was calculated by the Pearson correlation between each gene and the module eigengene. We further used the (MCODE) plugin to identify the most densely connected core subnetwork in the whole module network. MCODE is a graph theoretic clustering algorithm based on vertex weighting (34). Local neighborhood density and outward traversal from a locally dense seed protein are analyzed to define and select the most densely connected core subnetwork in the PPI network. Key parameters were set as default as follows: degree cutoff = 2, node score cutoff = 0.2 and K-core value = 2.

### Protein-protein network (PPI) construction

The Search Tool for the Retrieval of Interacting Genes (STRING) database (35) was used to build PPI networks in the selected gene module. This database provided information about known and predicted protein interactions based on regulation, correlation or protein binding validated in Co- IP and other experiments. STRING analysis results were used to construct the PPI network in the Cytoscape software v.3.9.1.

### CSN analysis

To explore the gene associations at the single-subject level, we used MATLAB software to construct the CSN (19) of gene associations for each individual subject in the RNA-seq data. Based on the expression values of genes X and Y in different subjects (**Figure S1A**), the CSN method constructed a scatter diagram in which each dot represented an individual subject, x-axis shows the expression values of gene X, and y-axis shows the expression values of gene Y for cell k. The number of dots (i.e., cell number) in the blue, red and intersection green boxes is denoted as n x (k), n y (k) and n xy (k) respectively. n was the total subject number in the scatter diagram. n x (k) = n y (k) = 0.1n was set as default. The coefficient 0.1 denotes the box size (blue and red boxes). In cell k, the statistic ρ*xy* (k) is used to assess the inter-relationships (edges) among gene x and gene y (Equation 1).


(1) $${\hat{\rho }}_{\text{xy}}^{{\;}^{\left(\text{k}\right)}}=\frac{\sqrt{\text{n}-1}\cdot \left(\text{n}\cdot {\text{n}}_{\text{xy}}^{{\;}^{\left(\text{k}\right)}}-{\text{nx}}^{\;\left(\text{k}\right)}\text{ny}{\;}^{\left(\text{k}\right)}\right)}{\sqrt{\text{nx}{\;}^{\left(\text{k}\right)}\text{ny}{\;}^{\left(\text{k}\right)}\left(\text{n}-\text{nx}{\;}^{\left(\text{k}\right)}\right)\left(\text{n}-\text{ny}{\;}^{\left(\text{k}\right)}\right)}}$$

### LASSO analysis

To select predictive features of osteoporosis risk in postmenopausal and premenopausal patients with low and normal BMD, genes in the core subnetwork were used to perform the LASSO analysis using the glmnet R package (20). Coefficients of unimportant variables were penalized to zero and important variables were retained with LASSO method. The retained predictors were then utilized to develop a binary logistic regression model for scoring osteoporosis risk. We used the area under the receiver operating characteristic (ROC) curve (AUC) to determine the discriminative ability of the model. Then we test the module significant by the “roc.area” function in R software (36). The Youden index was calculated in the pROC R package and used to determine the best ROC cutoff value (37).

## Results

### Integrated analysis identified OBCs and bone microenvironment cells

**Figure 1:**
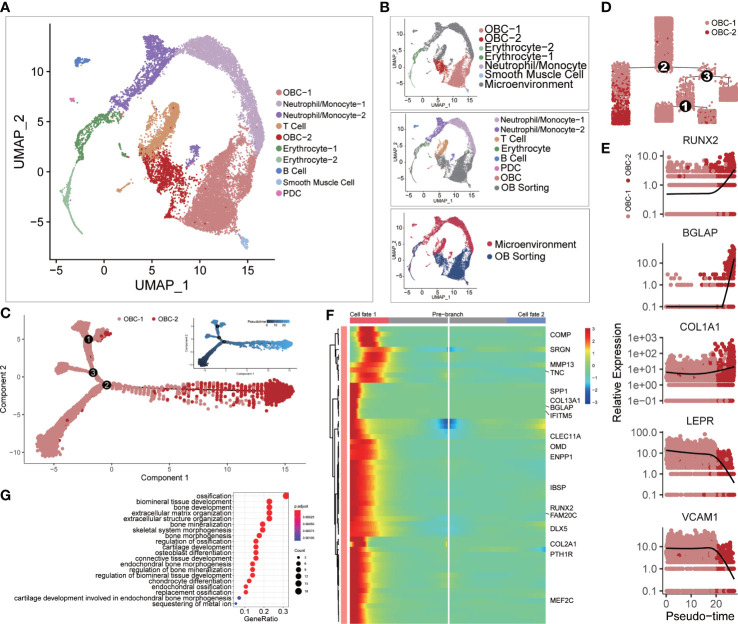
Single-cell clustering analysis. **(A)** Single-cell clustering results after CCA integration analysis. **(B)** The upper panel shows cell cluster information of microenvironment cells before integration analysis using the same clustering layout in panel **(A)** The middle panel shows cell cluster information of OB sorting dataset before integration analysis using the same clustering layout. The lower panel markers the source of each cell. **(C)** Cell developmental trajectory inference of OBC-1 and OBC-2. The upper-right trajectory plot indicates the direction of pseudotime. **(D)** Cell lineage relationships in panel **(C)**. **(E)** Expression levels (log-normalized) of indicated genes with respect to their pseudotime coordinates. The x-axis indicates the pseudotime, while the y-axis represents the log-normalized gene expression levels. Black lines depict the LOESS regression fit of the normalized expression values. **(F)** Continuum of up regulated-genes in cell fate 1 around branch point 2 in panel **(D)** Cell fate 1 is correlated to the left branch after branch point 2 in panel **(D)** Marked names of ossification-related genes. **(G)** GO analysis results of up regulated-genes in cell fate 1.

We integrated two scRNA-seq datasets of the femur head-derived bone tissue from a 31-year-old Chinese male subject before osteoblast isolation (Microenvironment dataset (11)) and after osteoblast isolation (OB sorting dataset (10)) through FACS (**Figure 1A**). The microenvironment dataset included seven original cell clusters before CCA analysis (**Figure 1B**, top): Neutrophil/Monocyte-1, Neutrophil/Monocyte-2, T cell, Erythrocyte, B cell, plasmacytoid dendritic cell (PDC) and OBC. The OB sorting dataset included six original cell clusters (**Figure 1B**, middle) before CCA analysis: OBC-1, OBC-2, Erythrocyte-1, Erythrocyte-2, Neutrophil/Monocyte and smooth muscle cell. After integration, the same type of cells such as OBC, erythrocyte and neutrophil/monocyte from the two different datasets clustered together, while the unique cell types like T cell, B cell and PDC in the microenvironment dataset and the smooth muscle cell in the OB sorting dataset were separated in the CCA UMAP plot as expected (**Figure 1B**, bottom; **Figure 1A**). However, some cell clustering results were unexpected and may not be sensible due to batch or sample effects before CCA (**Figure S2A**). For example, OBC cells in these two datasets were completely separate. In addition, Some B cells were mixed with the smooth muscle cell cluster. So CCA is needed in the integration of these two datasets. Differentially expressed gene (DEG) analysis identified top 10 DEGs in these 10 integrated cell clusters (**Figure S1B**). Cell type annotation was based on the expression patterns of recognized cell-type markers (**Figure S2B**).

**Figure 2:**
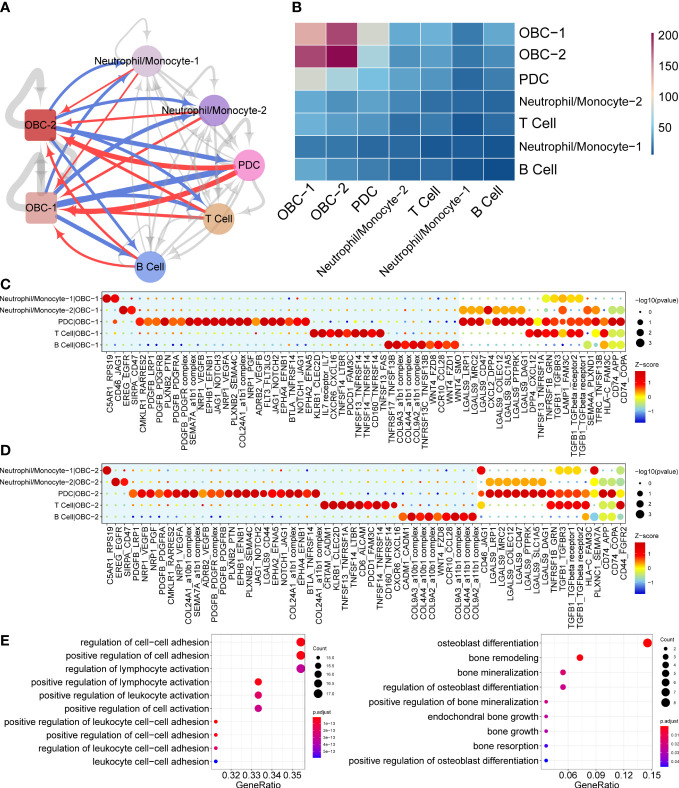
Cell communication results based on CellphoneDB. **(A)** Cell communication network of OBC and immune cell clusters. Width of edge represents the number of significantly differentially expressed L-R pairs. Edge direction is from ligand to receptor. Red edges are from immune cells to OBCs. Blue edges are from OBCs to immune cells. **(B)** Heatmap of significantly differentially expressed L-R pairs numbers between two cell clusters. **(C)** Dot plots show significantly differentially expressed L-R pairs between each immune cell type to OBC-1. Unique L-R pairs in the blue background are only significantly differentially expressed in one cell pair. **(D)** Dot plots show significantly differentially expressed L-R pairs between each immune cell type to OBC-2. Unique L-R pairs in the blue background are only significantly differentially expressed in one cell pair. **(E)** GO enrichment analysis results of ligands (left) and receptors (right) in significant L-R pairs in panels **(C, D)**.

For different subclusters of OBC, cell trajectory inference analysis results showed that OBC-2 was the late-stage cell compared with early-stage subcluster OBC-1 (**Figures 1C, D**). OBC-1 also highly expressed BMSCs markers such as LEPR (38) and VCAM1 (39); while OBC-2 highly expressed osteogenic markers such as RUNX2, BGLAP and COL1A1 (40, 41) (**Figure 1E**). A branch heatmap (**Figure 1F**) of the branch point 2 in **Figures 1C**, **2D** further showed a tendency toward up-regulation of ossification related genes (**Figure 1G**) in cell fate 1 (late-stage cell fate, left branch after branch point 2 in **Figure 1D**). So, we defined OBC-1 as early-stage OBC and OBC-2 as late-stage OBC based on these results.

### CellPhoneDB analysis revealed L-R interactions between OBCs and bone microenvironment immune cells

Cell phone communication network predicted different potential L-R interactions between OBC-1/OBC-2 and various immune cells including Neutrophil/Monocyte-1, Neutrophil/Monocyte-2, PDC, T cell and B cells in the bone microenvironment (**Figures 2A, B**). Dot plots showed significantly differentially expressed L-R pairs between each immune cell type with OBC-1 (**Figure 2C**) or OBC-2 (**Figure 2D**). Unique L-R pairs depicted in the blue background were only significantly differentially expressed in one cell pair (**Figures 2C, D**). Compared with any other immune cells, PDC cells have more unique L-R pairs in both immune cell-OBC-1 (**Figure 2C**) and immune cell-OBC-2 cell pairs (**Figure 2D**). This result suggested PDC cells have unique functions regarding L-R communications with OBCs. We next performed GO enrichment analysis of the ligand genes from immune cells (**Figure 2E**, left) and receptor genes from OBC-1 and OBC-2 (**Figure 2E**, right) in significant L-R pairs identified in the cellphoneDB analysis respectively. Receptor genes of OBC which were enriched in osteoblast differentiation and bone mineralization related terms (**Table S3**, **Figure 2E**) may play an important role in the functional response of immune related ligands in the bone microenvironment communication.

### Pathway-based cell interactions analysis between OBCs and bone microenvironment immune cells

**Figure 3:**
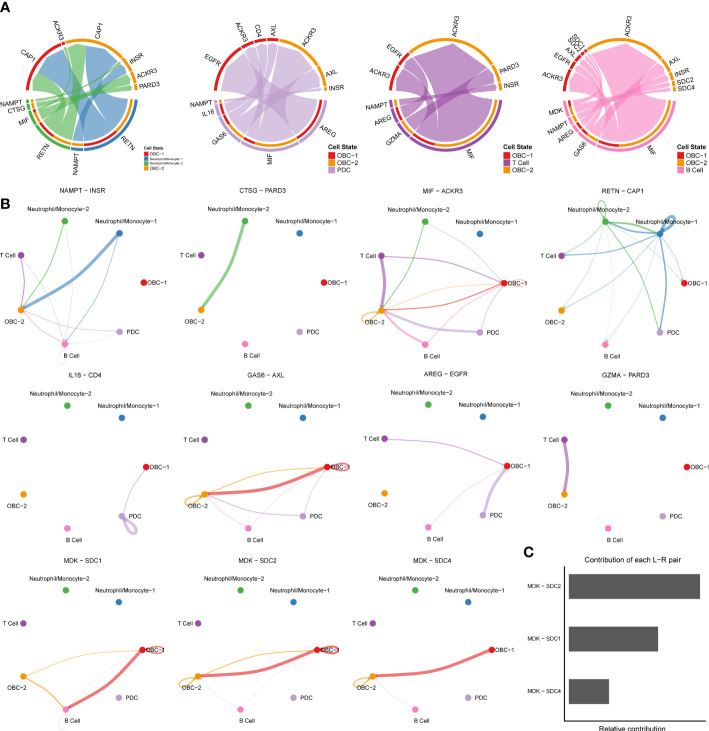
Cell communication results based on CellChat. **(A)** Chord diagrams of immune cell-OBC communications. Width of edge represents the interaction strength. Thicker edge line indicates a stronger signal. Edge direction was from ligand to receptor. **(B)** Circle plots of L-R pairs between different cell groups. Width of edge represents the interaction strength. Thicker edge line indicates a stronger signal. **(C)** Relative contribution of each L-R pair to the overall communication network of MK signaling pathway.

**Figure 4:**
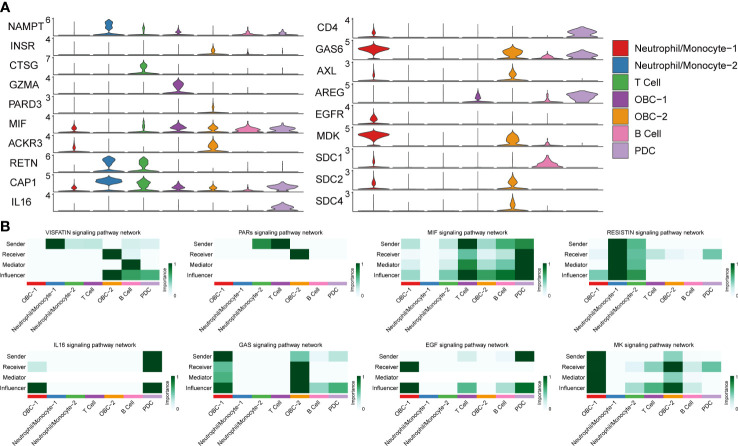
L-R Expression and signaling networks. **(A)** Expression levels of L-R genes in **Figure 3B**. **(B)** Heatmap shows the relative importance of each cell cluster based on the computed network centrality measures of different signaling networks.

We used CellChat to perform pathway-based L-R interactions analysis between OBCs and bone microenvironment immune cells. RETN-CAP1 (RESISTIN pathway) was the highest expressed interaction between Neutrophil/Monocyte-1 to OBC-1 and OBC-2. AREG-EGFR (EGF pathway) was the highest expressed interaction between PDC to OBC-1. MIF-ACKR3 (MIF pathway) was the highest expressed interaction between PDC to OBC-2, T cell to OBC-1, T cell to OBC-2, B cell to OBC-1 and B cell to OBC-2 (**Figure 3A**, **Table S4**). The communication network produced with CellChat further showed L-R pair expression levels across all different cell types (**Figure 3B**). MDK-SDC1/2/4 gene pairs were only involved in B cell-OBC interactions instead of any other immune cell-OBC interactions; MDK-SDC2 was the only interaction that was shared by both B cell-OBC-1 and B cell-OBC-2 cell pairs among MDK-SDC1/2/4 (**Figures 3A, B**, **Table S4**). As the SDC gene family has been reported to control apoptosis of OBC, we calculated the relative contribution of MDK-SDC1, MDK-SDC2, MDK-SDC4 among the MK pathway, and further found MDK-SDC2 was the most important contributor in this pathway (**Figure 3C**). The most important contributor was inferred to have potentially important biological contributions to the overall signaling pathway. A violin plot of all involved genes in the CellChat analysis also showed SDC2 expressed in both OBC-1 and OBC-2 clusters, while SDC1 and SDC4 were only expressed in OBC-1 and OBC-2, respectively (**Figure 4A**).

Based on different cell types’ L-R pair expression profiles (**Figures 3**, **4A**), we performed a variety of quantitative network measure analyses in an unsupervised manner provided by CellChat. Pathway heatmap showed the relative importance of each cell group in four different roles including sender, receiver, mediator and influencer (**Figure 4B**). For example, betweenness analysis showed B cell was the dominant mediator in VISFATIN signaling pathway, suggesting its role as a gatekeeper in this communication network; OBC-1 was a prominent influencer controlling the communications in IL6 signaling pathway confirmed by network centrality analysis (**Figure 4B**).

**Figure 5:**
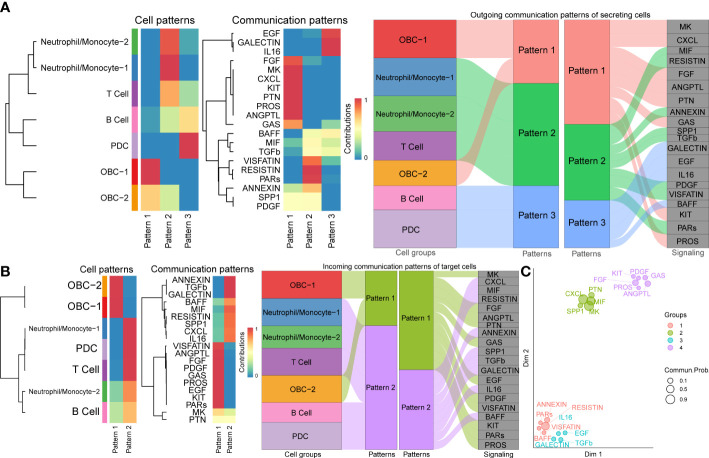
Cell communication patterns. **(A)** The inferred outgoing communication patterns of cells (Left plot). The inferred outgoing communication patterns of pathways (Middle plot). The correspondence between the inferred latent patterns of cell clusters and signaling pathways (Right plot). **(B)** The inferred incoming communication patterns of cells (Left plot). The inferred incoming communication patterns of pathways (Middle plot). The correspondence between the inferred latent patterns of cell clusters and signaling pathways (Right plot). **(C)** Projecting signaling pathways according to their functional similarity. Dot size is proportional to the overall communication probability. Different colors represent different pathway groups.

To explore how multiple cell groups and signaling pathways coordinate to function, we identify the global communication patterns that connect cell groups with signaling pathways either in the context of incoming signaling (treating cells as receivers) or outgoing signaling (treating cells as senders). This analysis uncovered three patterns for outgoing signals (**Figure 5A**) and two patterns for incoming signals (**Figure 5B**) that may coordinate with each other within the corresponding cell groups. The communication patterns of secreting cells (**Figure 5A**) show that outgoing immune signaling is dominated by Pattern 2 (Neutrophil/Monocyte-1, Neutrophil/Monocyte-2, T Cell) and Pattern 3 (B cell, PDC). Pattern 2 included bone related signaling pathways such as TGFβ, while Patterns 3 include bone related signaling pathways such as EGF. On the other hand, the incoming OBC signaling was characterized by Pattern 1 (**Figure 5B**), which represented multiple pathways such as EGF and PDGF. By identifying poorly studied pathways that group together with other well-known bone function related pathways like TGFβ, MK, MIF (42–46) in the functional similarity grouping analysis, these results showed predicted putative bone related functions of the former pathways (**Figure 5C**).

### Co-expression network identification of genes associated with monocyte subtypes

From L-R interaction analysis, we identified comprehensive communication patterns between immune cells and OBCs in local bone microenvironment. To further explore the systemic osteoimmunology communications in peripheral circulation, we investigated circulating immune cells’ association with bone health (1, 2). We selected monocyte for further analysis partially because it was also the sole source of osteoclast precursors in adult peripheral skeleton. First, we performed RNA-seq of peripheral blood monocyte from 944 male subjects (age:20-64). Second, we used the subset of 4 monocyte clusters (Mono1, Mono2, Mono3, Mono4) from a public scRNA-seq dataset (24) of human peripheral blood mononuclear cells to characterize monocyte subtype compositions by deconvolution analysis (30). The output was the cell proportion matrix of four monocyte subtypes for each sample from the formal bulk RNA-seq data of peripheral blood monocyte. Next, we performed WGCNA analysis to identify potential gene co-expression networks which were associated with BMD or certain monocyte subtypes.

**Figure 6:**
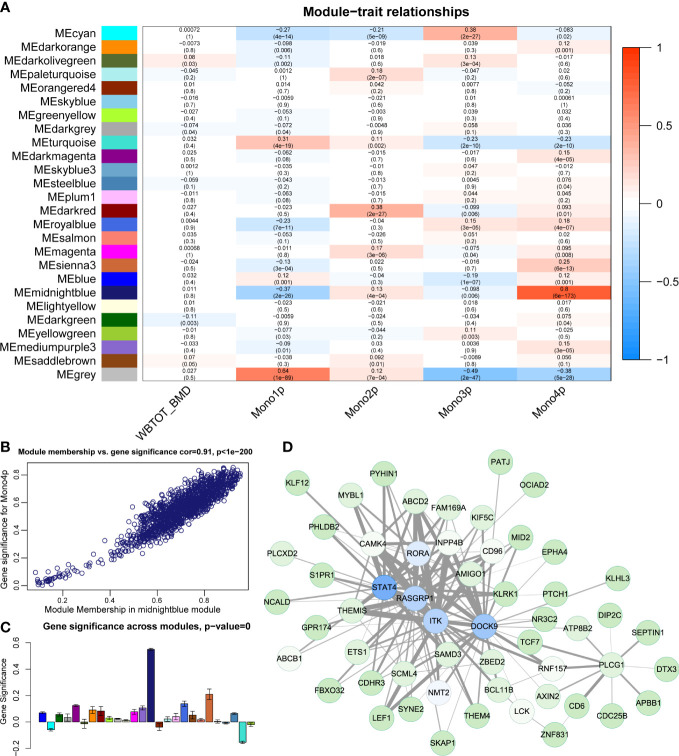
Construction of gene correlation modules. **(A)** Module-trait relationship heatmap. Each row indicates a module eigengene, and each column presents cell subtype proportion. The corresponding correlation and P value have been marked. **(B)** Scatter plots of module membership vs. gene significance in midnightblue module. **(C)** Gene significance of Mono4 proportion crosses modules. **(D)** Gene member network of midnightblue module. Top 150 gene connections (by topological overlap) among the top 100 hub genes (by kME) are shown in the network.

WGCNA identified 25 distinct functional modules (**Figure S3A**) in the RNA-seq data of PBMs. The midnightblue module was identified to have the highest correlation coefficient between its eigengene with the proportion of Mono4 (**Figure 6A**). However, no module showed a significant correlation (correlation coefficient > 0.7) with WB-BMD (**Figure 6A**). The high correlation coefficient between gene significance (reflecting how strongly the module gene expression values correlate with a certain cell type) vs. module membership (reflecting how strongly the module gene expression correlates with the module eigengene) further supports the significant association of the midnightblue module with Mono4 proportion (**Figure 6B**). In addition, the midnightblue module also had the highest gene significance score across modules (**Figure 6C**), indicating that the association of Mono4 proportion was specific in this gene module. Top 150 gene connections (topological overlap based on co-expression) among the top 100 hub genes (genes with the highest kME) of the midnightblue module were shown in the gene member network (**Figure 6D**).

Next, we performed function enrichment analysis of midnightblue module genes in the KEGG and Wiki databases. The enrichment results showed genes in the midnightblue module were significantly enriched in TGFβ signaling pathway and TGFβ receptor signaling in skeletal dysplasias (**Figure S3B**). As TGFβ signaling pathway was also involved in Neutrophil/Monocyte related incoming and outgoing patterns in our pathway-based L-R analysis, we further used Metascape to explore the relationship of TGFβ signaling pathway groups with other term groups. The results showed TGFβ signaling pathway related midnightblue module genes were widely associated with other skeletal related term groups represented by BMP signaling pathway and skeletal system development (**Figure S3C**).

### CSN analysis revealed the potential BMD protective effects of the core subnetwork of the midnightblue module in older males

**Figure 7:**
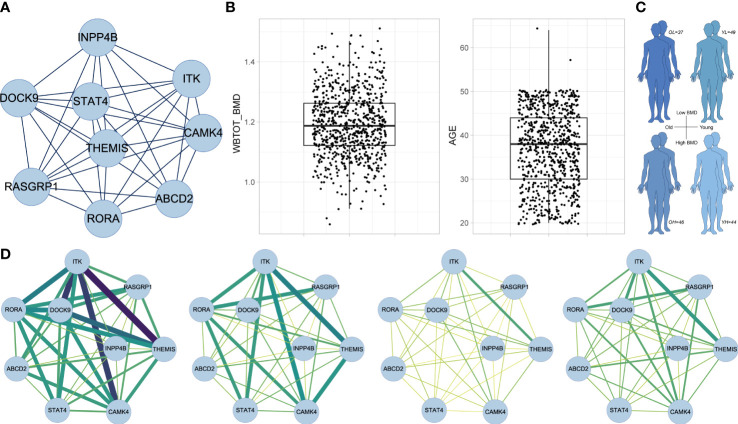
CSN analysis results in male subjects. **(A)** The subnetwork screened by MCODE. MCODE score = 8.5. **(B)** Box plots of whole-body BMD level (left) and subject age (right). **(C)** Age and BMD information of four subject groups. **(D)** CSN analysis results of subnetwork gene in OH, OL, YH and YL group. Edge represents mean connection score in each cell type from low (yellow/thin) to high (darkgreen/thick).

MCODE extracted a core subnetwork of the midnightblue module (**Figure 7A**) consisting of nine hub genes (MCODE score = 8.5) within the gene member network (**Figure 6D**). PPI analysis showed the protein interaction information of proteins of nine hub genes and other genes in the gene member network (**Figure S1C**). We next performed the CSN analysis to explore the correlation of gene interactions in the core subnetwork with BMD by using the RNA-seq data of PBMs from male adults. First, samples were categorized into quartiles (Q1, Q2, Q3, and Q4) according to BMD and age respectively (**Figure 7B**). We defined four sample groups (**Figure 7C**) as OH (oldest quartile in age and highest quartile in BMD), OL (oldest quartile in age and lowest quartile in BMD), YH (youngest quartile in age and highest quartile in BMD) and YL (youngest quartile in age and lowest quartile in BMD) group. Then CSN networks of nine hub genes were constructed in each group respectively. We observed that the hub genes were strongly interconnected only in the OH group. While other groups especially YH and YL groups showed weak interconnections in the network (**Figure 7D**). No significant correlation was found in Mono4 proportions with BMD levels, and no significant differences were found in Mono4 proportions in OH and OL groups. These results further supported that the effects of core subnetwork extracted from Mono4 were mainly based on gene interactions, instead of Mono4 proportions. The specific strong connections in the OH group suggested that high interactions of this subnetwork may be involved in the BMD specific protective effect in older males.

### Construction of osteoporosis risk prediction model within the core subnetwork of the midnightblue module

**Figure 8:**
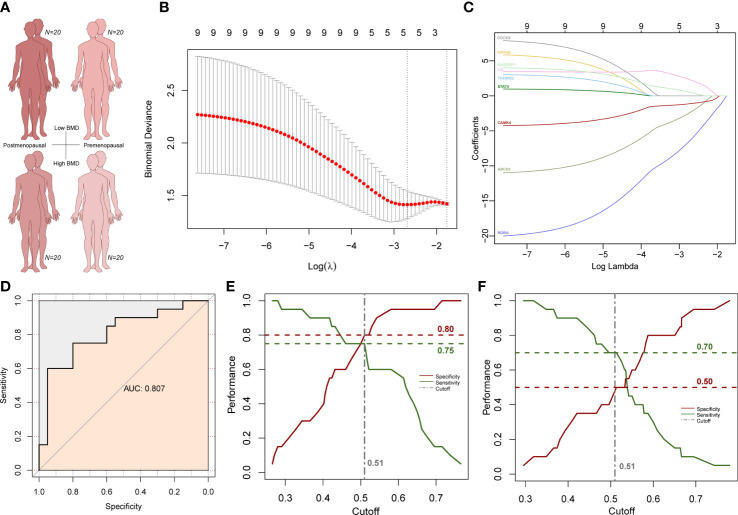
LASSO analysis results in female subjects. **(A)** Age and BMD information of four subject groups. **(B)** Subnetwork gene expression features selection in the LASSO model. **(C)** Coefficient curves of subnetwork genes. **(D)** ROC curves of the prediction model. **(E)** Cutoff selection based on specificity and sensitivity performance in postmenopausal group. **(F)** Specificity and sensitivity performance under the same cutoff value in the premenopausal group.

To explore whether the above subnetwork is also related to different BMD levels in older females, we selected predictive features within the subnetwork using LASSO method to predict osteoporosis in postmenopausal patients with low and normal BMD (**Figure 8A**). We used postmenopausal female osteoporosis data as the training dataset and premenopausal female osteoporosis data as the validation group. Five genes remained after the LASSO feature selection, and the formula for the osteoporosis risk score (ORS) was established as follows: ORS = 33.991 - 2.272 * ABCD2 - 6.201* RORA + 2.237 * ITK - 1.100 * CAMK4 + 0.466 * RASGRP1 (**Figures 8B, C**). The AUC was used to value the formula’s ability of prediction. The ORS was reliable for predicting osteoporosis as AUC is as high as 0.807 (**Figure 8D**). The ORS module is also statistically significant with a p-value at 0.00029. Then, Youden index was used to adjust the cutoff value and the classification result of ORS model. The Youden index analysis showed the best cutoff value was 0.51 (specificity = 0.8, sensitivity = 0.75; **Figure 8E**). The higher ORS score is corresponding to lower osteoporosis risk and higher BMD level. Next, we used the premenopausal female osteoporosis data to validate this model. Although the performance was not as good as the training dataset, the specificity was 0.50 and the sensitivity was 0.70 in this validation dataset (**Figure 8F**). These results further suggested the specific higher correlation of this subnetwork with higher BMD in older females, which was also consist with its strong correlation in older males with higher BMD.

Then we compared two known gene panels to our module (47–49). 29 BMD-associated gene were included in the first gene panel which were defined in the Dubbo Osteoporosis Epidemiology Study with 557 men and 902 women (47). AUC score of this gene panel is 0.775 in the LASSO analysis. Thirteen fracture risk-associated gene were included in another gene panel which were defined in a genome-wide meta-analysis with 31,016 cases and 102,444 controls (48, 49). AUC score of this gene panel is 0.787 in the LASSO analysis (48, 49). So, both known gene panels were less predictable with lower AUC scores less than 0.8. These results indicate the ORS model is efficient and specific in osteoporosis risk prediction.

## Discussion

In the current study, we performed cell communication analysis between OBCs and immune cells by integrating two scRNA-seq datasets generated from the same sample: a 31-year-old man who underwent hip replacement surgery. To further explore the bone health-related functions of immune cells, we used CSN and LASSO analysis in male and female subjects respectively, to reveal the potential role of a gene subnetwork associated with Mono4 which was identified by WGCNA.

Cell communications in the bone microenvironment are known to be important for bone homeostasis (1, 2, 6, 7). Receptors in the significant L-R interactions provided by CellPhoneDB were enriched in multiple bone related terms such as osteoblast differentiation and bone mineralization. For example, previous research has reported that Jagged1 was expressed concomitantly with Notch1 in maturating osteoblastic cells during bone regeneration (50). Interaction of Jagged1 and Notch1 was involved in enhancing BMP2-induced osteoblastic differentiation (50). Jagged1 and Notch2 were similarly localized in mesenchymal cells and regulated both endochondral and intramembranous bone regeneration (51). Our research further showed these JAG1-NOTCH interactions may also play an important role in PDC-OBC cell communication in the bone microenvironment. Active TGFβ1 release from cleaved LAP by osteoclastic bone resorption induced enrichment of osteoprogenitor in the bone resorption lacunae (42). Our results further showed that bone microenvironment immune cells such as Neutrophil/Monocyte-1, PDC and T cell may also be the important source of TGFβ1 to active TGFβ receptor-1/2 in both early-stage and late stage OBC. Compared with any other immune cells, PDC cells have more unique L-R pairs in both immune cell-OBC-1 (**Figure 2C**) and immune cell-OBC-2 cell pairs (**Figure 2D**). In addition, FLT3-FLT3LG and JAG1-NOTCH3 were unique PDC-OBC-1 cell pairs in the PDC-OBC communications; while LGALS9-CD44, COL24A1-a10b1 complex and COL24A1-a11b1 complex were unique PDC-OBC-2 cell pair in the PDC-OBC communications. Moreover, Jagged1 and Notch3 were up-regulated during osteogenic differentiation of human bone marrow-derived mesenchymal stromal cells *in vitro* and bone healing period of murine tibial fracture *in vivo* (52). These results suggested PDC cells have unique functions regarding L-R communications with early and late-stage OBCs.

Pathway based CellChat analysis showed NAMPT-INSR (VISFATIN pathway) was a common L-R pair among all immune cell types to OBC-2. Nampt plays a critical role in osteoblast differentiation through epigenetic augmentation of Runx2 transcription (53). Nampt induced activation of insulin signaling by INSR influenced not only postnatal bone acquisition but also of bone resorption (54, 55). The interactions through different immune cell types to the late-stage OBC regarding NAMPT-INSR suggested a complex regulatory mechanism may involve in this functional L-R pair. There are also unique bone related L-R pairs that only showed in certain cell pairs such as MDK-SDCs in B cell-OBC interactions (43–45). SDCs are proteoglycans that act as signaling molecules. Previous research showed that SDC2 is involved in the control of OBC apoptosis (44). As CellChat analysis also supported MDK-SDC2 was the most important contributor in the corresponding MK pathway, further investigation may focus on the functional role of SDC2 based on this interaction.

Circulating immune cells also influence bone states (56, 57). Aging and impaired circulating monocytes were chemotactic to bone lesions. So, we chose peripheral blood monocytes, the sole source of osteoclast precursors in peripheral bone [14, 15] to investigate their potential association with BMD. First, we found a Mono4 related gene module (midnightblue module) by deconvolution and WGCNA analysis. Mono4 expressed two kinds of signature genes, i.e., classical monocyte signature genes (e.g., TLR2, CTSD, NLRP3) and distinctive cytotoxic signature genes (e.g., PRF1, GNLY, CTSW), resembling previously reported “natural killer dendritic cells” (24). Natural killer dendritic cells provide a link between innate and adaptive immunity, and involve in antigen presentation, cytotoxic and antitumor activities (58, 59). However, the specific function of the Mono4 cell subtype is still poorly understood. Genes in the midnightblue module were highly expressed in this cell subtype or regulated by Mono4 in the whole monocyte group. GO analysis showed the midnightblue module genes were enriched in TGFβ pathway. Metascape further showed the TGFβ pathway-related genes also have correlations with other bone function related terms such as skeletal system development and BMP signaling pathway. RORA was one of the nine hub genes in the midnightblue module identified by MCODE. Previous studies have examined RORA’s function in bone metabolism (60). RORA-deficient mice exhibit abnormalities in bone formation and bone tissue maintenance (60). In the CSN analysis results, hub genes showed a high association in OH group. These association edges may come from co-expression, gene regulation, alternative splicing and so on. Strong interactions among hub genes suggested their unique function in maintaining high BMD in the older male at the gene interaction perspective. LASSO analysis showed hub genes can predict low/high BMD group in postmenopausal female subjects, which further underline their potential function in this population. There are some similarities and differences between the osteoimmunology communication results in older male and postmenopausal female samples. For example, ITK showed the highest association within the CSN network of HO group, and it also has the highest coefficient in the osteoporosis risk score (ORS, high score is corresponding to low osteoporosis risk) formula of postmenopausal female. While the other CSN network gene with higher association in the HO group, DOCK9, is not a predictive feature for osteoporosis in postmenopausal females. These results showed that the function of DOCK9 might be more specific in postmenopausal females compared with the potentially general effect of ITK. So, pathological conditions and gender differences should be considered in the further osteoimmunology communication related research.

One limitation of the present study is that much of the research data used in the microenvironment cell communication analysis were obtained from one 31-year-old Chinese male patient. This sample size is limited in its ability to fully represent the general osteoimmunology pattern. Specifically, more samples from both healthy subjects and patients with bone disorder are needed to derive unbiased expression matrices for the downstream analyses of osteoimmunology communication. Despite this potential limitation, our results provide the first necessary and valuable insights into the predictive microenvironment osteoimmunology communication at the single cell level. Secretory factors produced by immune cells influenced osteoblast biology such as increased osteoblast activity, reduced viability and increased apoptosis (1, 2), which provided the evidence for the immune cells-osteoblast interactions’ substantial effect. Although direct cell contact is not required to exert this effect, increases in immune cell number within the close proximity of osteogenic cells could likely increase the likelihood of immune-osteogenic cell interactions. These insights on the predicted L-R interaction perspective may prove critical for the basic understanding of bone metabolism and pathophysiologic mechanisms associated with various bone disorders.

## Conclusion

In conclusion, our research revealed a comprehensive intercellular interactions landscape between microenvironment immune cells and OBCs. We also found a Mono4 related subnetwork to further strengthen the evidence of immune cells’ function in bone health in older males and postmenopausal females. Our results establish the foundations to investigate advanced mechanisms regarding both microenvironment and circulating immune cells’ impact on BMD and the related skeletal disorders such as osteoporosis and traits as well.

## Data availability statement

The scRNA-seq datasets from the 31-year-old male subject can be accessed with accession numbers under GSE169396 (Microenvironment dataset) and GSE147390 (OB sorting dataset) in GEO database. The female osteoporosis mRNA transcriptome array data can be accessed with accession number under GSE56815 in GEO database. The LOS dataset which was partly used in the current study are not publicly available due to the study is still on going, but are available from the corresponding author on reasonable request.

## Ethics statement

The studies involving human participants were reviewed and approved by The Institutional Review Boards of Tulane University. The patients/participants provided their written informed consent to participate in this study.

## Author contributions

SW was involved in the study conceptualization, methodology, data analysis, writing-original draft, and writing-review and editing. JG, CQ, YG, ZW, XL, YL, PH, XM, QZ, and HS were involved in the study methodology, and writing-review and editing. KV, FS, MS, and HX were involved in the study and writing-review and editing. HD and HS were involved in the study conceptualization, funding acquisition and writing-review and editing. All authors contributed to the article and approved the submitted version.

## Supplementary material

Supplementary Figure 1
**(A)** Each dot in the scatter plot represents an individual cell. X-axis represents the expression values of gene x, Y-axis represents the expression values of gene y. The red dot represents cell k. The number of dots in the yellow, green and intersection blue boxes near the red dot (cell k) are denoted as ${\text{n}}_{N}^{\left(k\right)}$ ${\text{n}}_{x}^{\left(k\right)}$ , ${\text{n}}_{y}^{\left(k\right)}$ and ${\text{n}}_{\text{xy}}^{\left(k\right)}$ respectively. **(B)** Relative gene expression level of top 10 most significant differentially expressed genes for each cluster. C. PPI network based on genes in . Yellow nodes represent Mono4 related subnetwork genes. Edge represents the evidence probability valued by combined score.

Supplementary Figure 2
**(A)** Single-cell clustering results before CCA integration analysis. **(B)** Expression of marker genes in each cell cluster.

Supplementary Figure 3
**(A)** The clustering dendrograms of 25 co-expression gene modules by different colors. Each gene is represented by one branch. **(B)** Function enrichment results of genes in the midnightblue module. **(C)** Pathway correlation analysis of TGFβ signaling pathway related midnightblue module genes. Metascape enrichment network visualization. Edges reflect the relatedness of two term clusters. Cluster annotations (left plot) or adjusted p values (right plot) are shown in different colors. Dot size represents gene number in each term.

Supplementary Table 1 Detailed characteristics of subjects for the PBMs RNA-seq analysis.

Supplementary Table 2 Complex information of all L-R pairs that are involved in the CellPhoneDB analysis.

Supplementary Table 3 Enriched genes in the bone function related GO terms correspond to **Figure 2E**.

Supplementary Table 4 Pathway information of each L-R pair in **Figure 3A**.
