# Supplementary figures and images for: Single-cell RNA sequencing reveals in vivo osteoimmunology interactions between the immune and skeletal systems
Source: Front Endocrinol (Lausanne). 2023 Mar 27;14:1107511. doi: 10.3389/fendo.2023.1107511 (PMC10083244; doi:10.3389/fendo.2023.1107511)

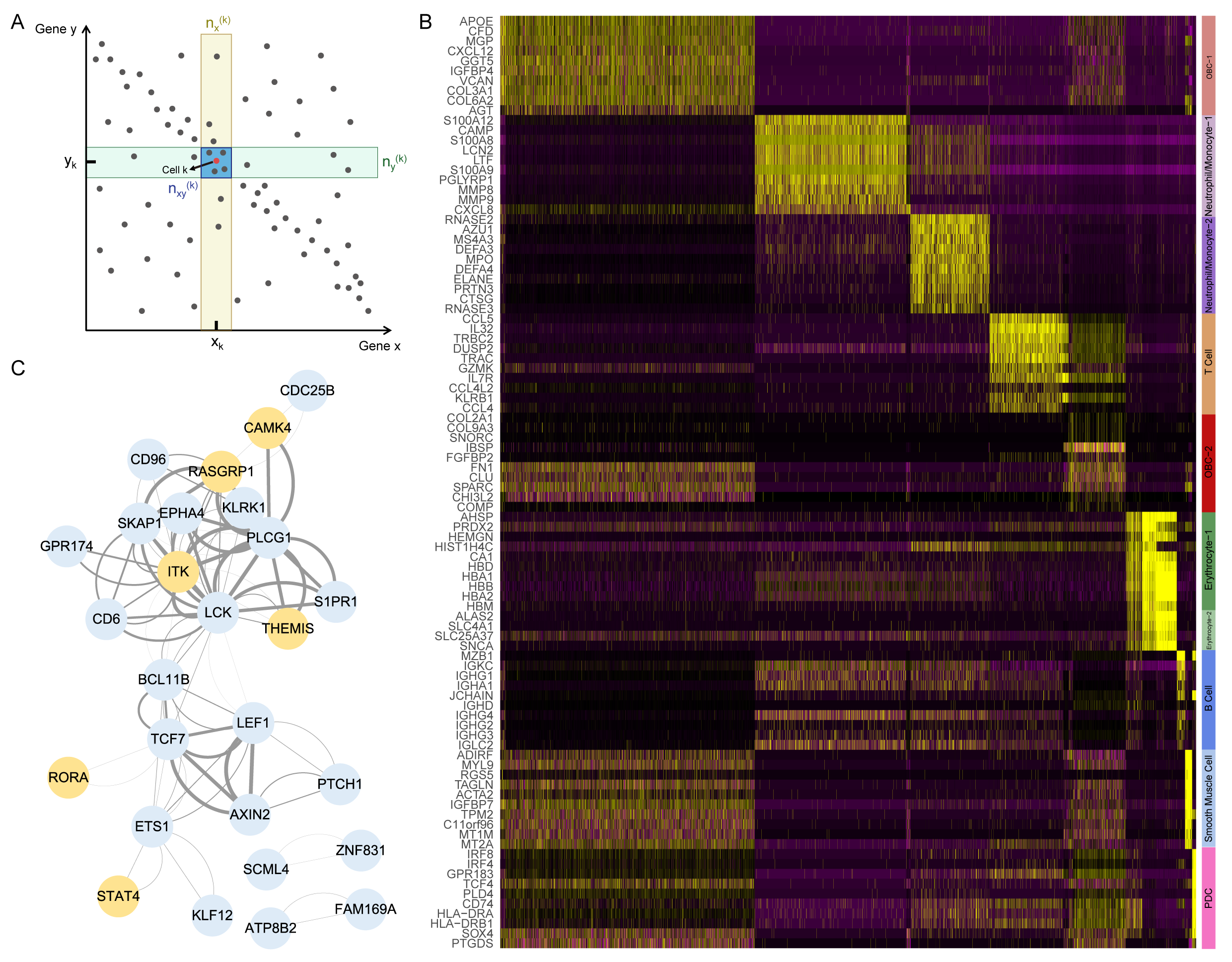

Supplement: Supplementary Figure 1 — (A) Each dot in the scatter plot represents an individual cell. X-axis represents the expression values of gene x, Y-axis represents the expression values of gene y. The red dot represents cell k. The number of dots in the yellow, green and intersection blue boxes near the red dot (cell k) are denoted as nN(k) nx(k) , ny(k) and nxy(k) respectively. (B) Relative gene expression level of top 10 most significant differentially expressed genes for each cluster. C. PPI network based on genes in . Yellow nodes represent Mono4 related subnetwork genes. Edge represents the evidence probability valued by combined score. [file Image_1.tif]

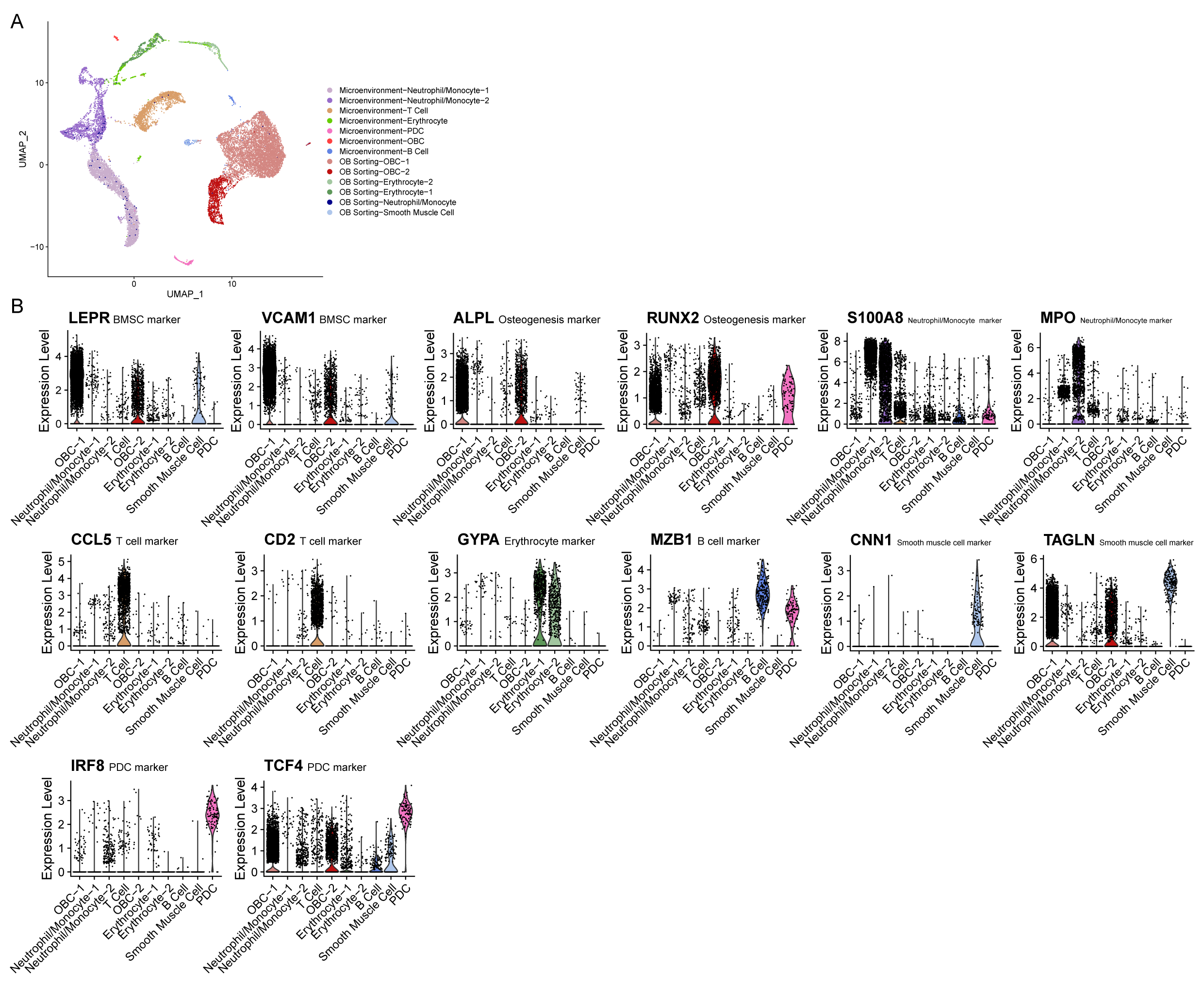

Supplement: Supplementary Figure 2 — (A) Single-cell clustering results before CCA integration analysis. (B) Expression of marker genes in each cell cluster. [file Image_2.tif]

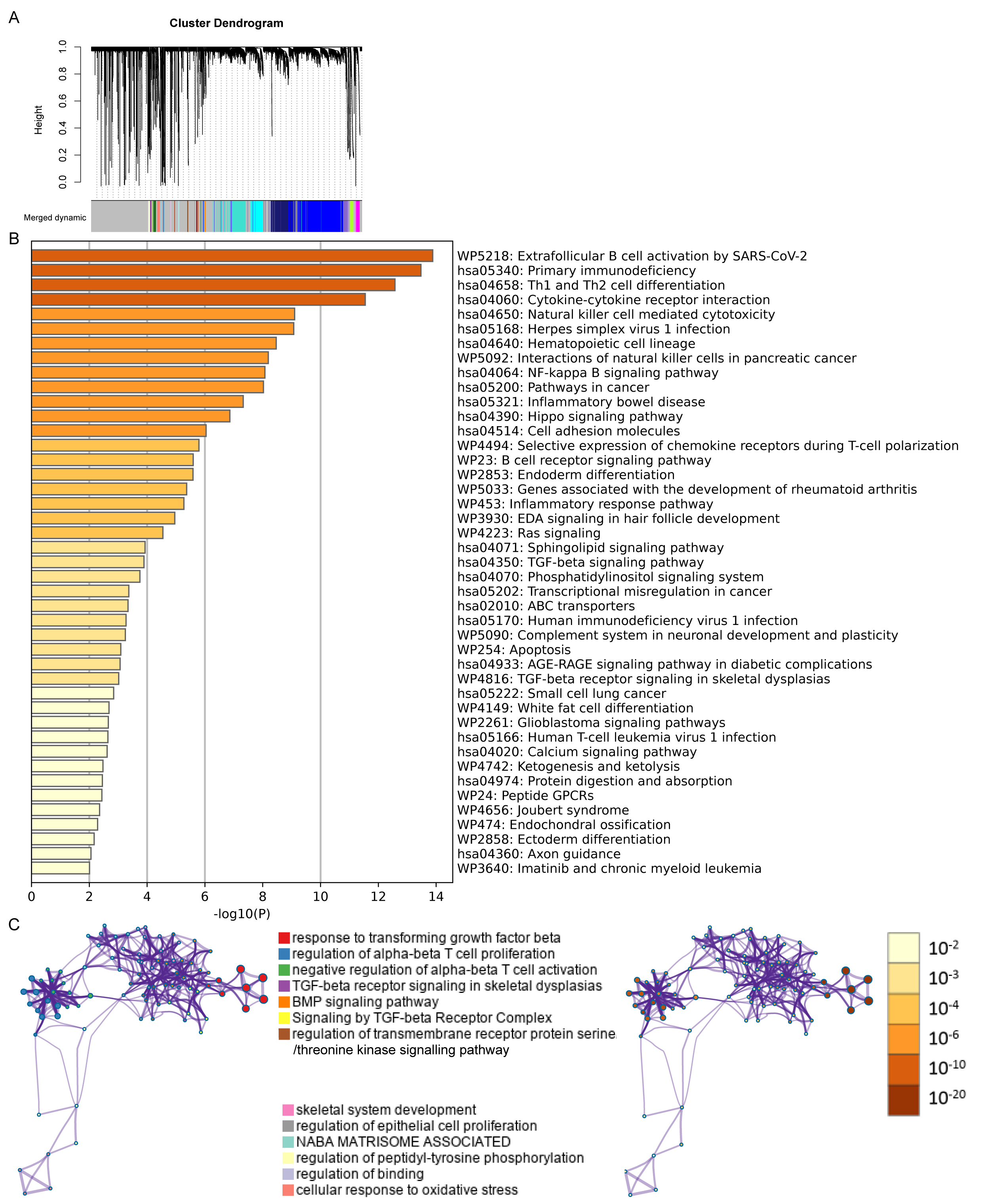

Supplement: Supplementary Figure 3 — (A) The clustering dendrograms of 25 co-expression gene modules by different colors. Each gene is represented by one branch. (B) Function enrichment results of genes in the midnightblue module. (C) Pathway correlation analysis of TGFβ signaling pathway related midnightblue module genes. Metascape enrichment network visualization. Edges reflect the relatedness of two term clusters. Cluster annotations (left plot) or adjusted p values (right plot) are shown in different colors. Dot size represents gene number in each term. [file Image_3.tif]
